# Editorial: Imaging Synapse Structure and Function

**DOI:** 10.3389/fnsyn.2016.00036

**Published:** 2016-12-15

**Authors:** George J. Augustine, Marc Fivaz

**Affiliations:** ^1^Lee Kong Chiang School of Medicine, Nanyang Technological UniversitySingapore, Singapore; ^2^Program in Neuroscience and Behavioral Disorders, Duke-NUS Medical SchoolSingapore, Singapore

**Keywords:** synapse, optical microscopy, electron microscopy, circuits, genetically-encoded biosensors, optogenetics

These are the glory days for imaging synapse structure and function. Thanks to recent advances in both optical and electron microscopy, it is now possible to image individual synapses with unprecedented spatial and temporal resolution. The parallel development of a wide range of genetically-encoded synaptic reporters enables all-optical recording of synaptic activity in genetically-defined neuronal populations. These engineering breakthroughs allow neuroscientists to interrogate the brain in ways that were inconceivable just a few years ago. The ability to image synaptic structure and activity in large functional circuits is beginning to yield key insights into how the brain stores, processes, and computes information. This research topic consists of eleven articles (methods, primary research papers, and reviews) that provide an overview of the latest developments in synapse imaging. Rather than attempting an exhaustive list of synaptic reporters and microscopy techniques, our collection emphasizes approaches that merge technical advances from diverse areas to extract a rich palette of novel information from individual synapses.

Watanabe presents a method that combines optogenetics and rapid freezing (Flash-and-Freeze) to visualize the synaptic vesicle (SV) cycle at the ultrastructural level with millisecond resolution Watanabe. This revolutionary approach revealed ultra-fast endocytosis of SVs at central synapses and neuromuscular junctions (Watanabe et al., 2013a,b) and promises to uncover many new kinetic aspects of synapse dynamics. Begemann and Galic review recent efforts to image neuronal preparations with both light and electron microscopy, with a series of hybrid techniques referred to as Correlative Light Electron Microscopy (CLEM). Jackson and Burrone describe the first genetically-encoded fluorescent reporter (sypHy-RGECO) that enables concurrent monitoring of calcium dynamics and SV fusion. sypHy-RGECO will undoubtedly be a powerful means of examining calcium triggering of SV exocytosis at the level of individual presynaptic boutons. Using similar probes, Tang et al. show that the mental disease gene DISC1 (Disrupted-In-Schizophrenia-1) accelerates SV exocytosis by facilitating calcium influx through N-type voltage-gated Ca^2+^ channels. Calcium transients at synapses are also shaped by both mobilization and sequestration of calcium by intracellular stores. Kwon et al. report on the latest advances in organelle-specific calcium sensors and review the contribution of the endoplasmic reticulum and mitochondria to calcium dynamics and synaptic transmission/plasticity.

Until recently, one major impediment to imaging of synaptic activity has been our inability to directly measure membrane potential with adequate signal/noise ratio. This is rapidly changing with the recent improvement of a wide range of genetically-encoded voltage indicators (GEVIs) that now are capable of monitoring both single action potentials and even subthreshold synaptic potentials, both *in vitro* and *in vivo* Nakajima et al. Three papers describe recent advances on the localization, dynamics and function of postsynaptic receptors and scaffolds. Using a combination of single-molecule tracking (uPAINT) and photoactivated localization microscopy (PALM), Li and Blanpied assess the diffusion properties of membrane proteins within the postsynaptic density (PSD). The same authors recently used localization microscopy to demonstrate the existence of transsynaptic nanocolumns that align the neurotransmitter release machinery to postsynaptic receptors (Tang et al., 2016a). Dosemeci et al. review a series of EM studies that reveal the presence of a dense lamina—the “pallium”—just beneath the core layer of the PSD, and discuss how translocation of signaling proteins and scaffolds in and out of the pallium may shape activity-induced changes in dendritic spines. In keeping with the theme of postsynaptic signaling, Dore et al. discuss evidence for metabotropic functions of NMDARs, based on time-resolved FRET and other imaging approaches. Finally, at the level of synaptic circuits, two reviews describe the use of genetically-encoded synaptic labels to trace neural circuits in a variety of different model systems, ranging from *C. elegans* to mammals (Hong and Park; Lee et al.).

Overall, we hope that the fine collection of papers contained within this research topic highlights a useful synapse imaging toolkit for the neuroscience community. The next big challenge in brain imaging will be to scale up these synaptic measurements to large ensembles of neurons to comprehend how circuits compute. This will require synaptic reporters that operate in a synaptically-relevant time scale (milliseconds), along with improved genetic targeting strategies, further advances in automated high-speed microscopy, and refined bioinformatics tools for analysis of the resulting large datasets.

## Author contributions

All authors listed, have made substantial, direct and intellectual contribution to the work, and approved it for publication.

## Funding

This work was supported by Singapore Ministry of Education grants MOE2015-T1-001-069 and MOE2015-T2-2-095 to GA and grant MOE2013-T2-1-053 to MF.

### Conflict of interest statement

The authors declare that the research was conducted in the absence of any commercial or financial relationships that could be construed as a potential conflict of interest.
